# Dynamic and self-biodegradable polysaccharide hydrogel stores embryonic stem cell construct under ambient condition

**DOI:** 10.3389/fbioe.2023.1169124

**Published:** 2023-05-11

**Authors:** Kuan Yang, Wei Wei, Li Ting Gao, Xin Yi Zhao, Zhenqi Liu, Jianhui Li, Haopeng Li, Hideyuki Miyatake, Yoshihiro Ito, Yong Mei Chen

**Affiliations:** ^1^ College of Bioresources Chemical and Materials Engineering, National Demonstration Center for Experimental Light Chemistry Engineering Education, Shaanxi University of Science and Technology, Xi’an, China; ^2^ College of Chemistry, Xi’an Jiaotong University, Xi’an, China; ^3^ Department of Surgical Oncology, Shaanxi Provincial People’s Hospital, Xi’an, China; ^4^ Second Affiliated Hospital of Xi’an Jiaotong University, Xi’an Jiaotong University, Xi’an, China; ^5^ Nano Medical Engineering Laboratory, RIKEN Cluster for Pioneering Research, Emergent Bioengineering Materials Research Team, RIKEN Center for Emergent Matter Science, Wako, Japan

**Keywords:** dynamic polysaccharide hydrogel, self-biodegradation, embryonic stem cell construct, storage, ambient condition

## Abstract

The proper microenvironment is critical for the storage and transportation of embryonic stem cells (ESCs). To mimic a dynamic 3D microenvironment as it exists *in vivo* and consider “off-the-shelf” availability reaching the destination, we proposed an alternative approach that allows for facile storage and transportation of stem cells in the form of ESCs-dynamic hydrogel construct (CDHC) under ambient conditions. To form CDHC, mouse embryonic stem cells (mESCs) were *in-situ* encapsulated within a polysaccharide-based dynamic and self-biodegradable hydrogel. After storing CDHC in a sterile and hermetic environment for 3 days and then transferring to a sealed vessel with fresh medium for another 3 days, the large and compact colonies retained a 90% survival rate and pluripotency. Furthermore, after transporting and arriving at the destination, the encapsulated stem cell could be automatically released from the self-biodegradable hydrogel. After continuous cultivation of 15 generations of retrieved cells, automatically released from the CDHC, the mESCs underwent 3D encapsulation, storage, transportation, release, and continuous long-term subculture; resumed colony forming capacity and pluripotency were revealed by stem cell markers both in protein and mRNA levels. We believe that the dynamic and self-biodegradable hydrogel provides a simple, cost-effective, and valuable tool for storing and transporting “ready-to-use” CDHC under ambient conditions, facilitating “off-the-shelf” availability and widespread applications.

## Graphical Abstract

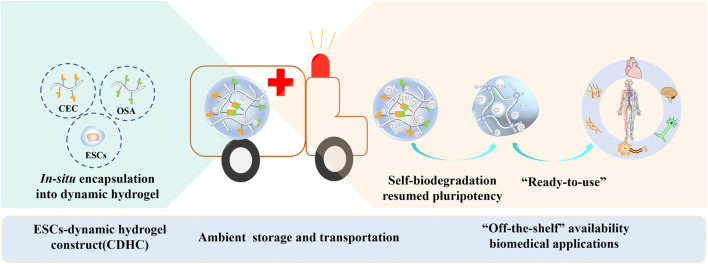

## Highlights

- ESCs-dynamic hydrogel construct (CDHC) could be constructed through *in-situ* encapsulation of embryonic stem cells (ESCs) into polysaccharide based dynamic and self-biodegradable hydrogel.
- Large and compact colonies developed after storing and transportation of CDHC under ambient conditions.
- Automatically released ESCs resumed colony forming capacity and pluripotency.
- Storage and transportation of “ready-to-use” CDHC under ambient conditions facilitats “off-the-shelf” availability and widespread biomedical applications.

To mimic the dynamic 3D microenvironment in vivo and consider “off-the-shelf” availability reaching the destination, we propose an alternative approach to produce ESCs-dynamic hydrogel construct (CDHC), simultaneously considering storage and transportation of stem cells under ambient conditions. The mESCs undergone 3D encapsulation, storage, transportation, automatical release and continuous long-term subculture, resumed colony forming capacity and pluripotency, revealing stem cell markers both in protein and mRNA levels.

## 1 Introduction

A suitable microenvironment is crucial for the storage and transport of embryonic stem cells (ESCs), as they are highly active and the pluripotency of ESCs is very sensitive to disturbance by various stimuli. ESCs encapsulation in hydrogels has enormous potential for biomedical applications, including cell-based medicine, cytotherapy, tissue engineering, regenerative medicine, drug screening, and genetic disease research (Xue et al., 2018; Fang et al., 2021). Pluripotent ESCs are derived from the inner cell mass of pre-implantation embryos and display the unique properties of infinite proliferation, self-renewal, and multidirectional differentiation (Fu et al., 2021; Hoang et al., 2022). Hydrogels, that mimic the extracellular matrix (ECM) microenvironment, possess high moisture content, excellent material transfer ability, and a soft tissue-like elastic modulus. These properties enable them to protect encapsulated cells from external force and immune rejection (Wang M. et al., 2022), at the same time, they facilitate cell survival and function by promoting the transport of oxygen and nutrients, while also expelling secreted molecules and metabolic wastes (Rosales and Anseth, 2016; Huang et al., 2017).

A crucial step before usage is necessary for the quick availability of various applications: efficient storage of cell-laden hydrogel and reliable preservation for sustaining ESCs pluripotency. However, there are still logistical and bioprocessing challenges that need to be addressed to preserve stem cells (Murray and Gibson, 2022; Zhao et al., 2022). Storage methods can significantly affect cell performances, especially ESCs with stress sensitivities (e.g., external force, chemical substances, and temperature change). Cryopreservation is used to full fill the tasks of long-term storage and long-distance transportation associated with several biological and technical issues, including harsh conditions, uneconomical facilities, and complex procedures (Liu M. et al., 2022; Liu Z. et al., 2022; Zhan et al., 2022). Therefore, the major bottleneck is the safe and efficient implementation of ESCs storage and transportation under ambient conditions (Jiang et al., 2017; Guo et al., 2022), as well as the widespread applications of “off-the-shelf” availability.

We consider that an alternate strategy through the creation of a “ready-to-use” ESCs-hydrogel construct (CDHC) may overcome these obstacles, which can be done through the storage of stem cells within dynamic hydrogel via *in-situ* encapsulating of cells under physiological conditions. Meanwhile, the storage of stem cells encapsulated in dynamic hydrogels with the properties of biocompatibility and self-biodegradability may allow generating cell-biomaterial construct, which potentially realizes automatic cell release for “off-the-shelf” availability. The strategy does not require uneconomic facilities and cumbersome operating procedures. This “off-the-shelf” associated with transportation between the sites of manufacture and the destinations facilitates to use of CDHC directly. Designing the platform to realize the above ideal is an ongoing challenge. Self-healing hydrogels crosslinked via dynamically reversible bonds have the features of restoring their integrated structures and functionalities after damage. Because dissociation and association or recombination of reversible interactions between polymer chains can reestablish the dynamic equilibrium of networks to self-repair damaged regions (Wei et al., 2014; Rizwan et al., 2021), these reversible features of dynamic hydrogels mimic the intrinsic structural dynamics of the ECM microenvironment and soft tissues (Wei et al., 2020; Lou and Mooney, 2022). While existing strategies focus on dynamic hydrogels as biomaterials for the biomedical field (Wei et al., 2014; Wei et al., 2015; Rosales and Anseth, 2016; Wei et al., 2016; Wei et al., 2020; Zheng et al., 2021; Li S. et al., 2022; Eftekhari-Pournigjeh et al., 2022; Wang et al., 2023), much less effort has been exerted to formally evaluate the storage and transportation potentials of cell-dynamic hydrogel construct in which ESCs are encapsulated in dynamic and self-biodegradable hydrogels.

Here, we propose a “ready-to-use” ESCs-hydrogel construct based on a dynamic polysaccharide hydrogel, that considers both the storage and transport of ESCs and the “off-the-shelf” availability for widespread applications. Because encapsulation of stem cells in polysaccharide hydrogels is an enabling technology for producing “off-the-shelf” cell-biomaterial constructs, permitting the automatic release of stem cells in the process of self-biodegradation of polysaccharide hydrogels while maintaining their viability and phenotype for biomedical applications and basic research.

In view of the fact that 1) ESCs can be conveniently encapsulated *in-situ* in dynamic hydrogel under physiological conditions by mixing cell suspension and cell culture medium solving polysaccharides. 2) Synergistic effect of self-biodegradation and reversible bonding can accommodate the drastic volume expansion of growing colonies 3D encapsulated in polymer networks. 3) Superb biocompatibility and self-biodegradability of polysaccharides and dynamic bonds ensure high quality of ESCs-hydrogel constructs and automatically released cells for “off the shelf” availability. We developed a facile and economical approach to produce ESCs-dynamic hydrogel construct simultaneously considering the storage and transportation of stem cells under ambient conditions, as well as “off-the-shelf” availability for automatically released cells to their destination.

We fabricated a self-healing polysaccharide-based CEC-l-OSA hydrogel (“l” means “linked-by”) crosslinked by Schiff base reaction with imide bonds, in which oxidized sodium alginate (OSA) and N-carboxyethyl chitosan (CEC) were used as biocompatible and biodegradable backbones for *in-situ* encapsulation of ESCs. It is demonstrated that CEC-l-OSA hydrogel significantly promoted clonal expansion and preserved good pluripotency of the encapsulated mESCs which were subjected to storage in a sealed vessel for 3 days and then transferred to a fresh medium for another 3 days. Furthermore, the mESCs automatically released from dynamic hydrogel sustained pluripotency during continuous cultivation for 15 generations (over 25 days) with cell passaging every 5 days. The synergistic effect of self-biodegradation and reversibly crosslinked polymer networks of the hydrogel can provide a dynamic microenvironment and spaces to accommodate the growth of mESCs clonal and retain pluripotency.

This approach is facile for realizing short-term cell storage and express transportation of stem cells between different institutions under ambient conditions. Most importantly, after arriving at the destination, the characteristics of the stem cells were not changed, and the CDHC can be directly used without any further treatment. Our findings highlight that dynamic and self-biodegradable hydrogel provides a versatile and practical tool of facile, economical, and reliable stem cell-biomaterial construct to support the storage and “ready-to-use” properties of ESCs, facilitating “off-the-shelf” availability and widespread applications in biomedical fields and research.

## 2 Materials and methods

### 2.1 Materials

Chitosan (CAS: 9012-76-4, degree of deacetylation 86%, Mw = 200,000 Da) was purchased from Tokyo Kasei Kogyo Co., Ltd. Sodium alginate (CAS: 9005-38-3, >350 mpa.s) was ordered from Alfa Aesar. Sodium periodate (CAS: 7790-28-5) was bought from Sigma-Aldrich and acrylic acid (CAS:79-10-7) was obtained from Aladdin Biochemical Technology (Shanghai, Co., Ltd). All chemical reagents were analytical grade and used without further purification.

### 2.2 Synthesis and characterization of CEC and OSA

CEC was synthesized through Michael’s addition reaction, and OSA was synthesized by oxidation of sodium periodate, according to our previously reported method (Wei et al., 2015). The degree of substitution of the amino group in CEC is 48%. The oxidation degree of OSA is 50% determined by hydroxylamine hydrochloride titration. The details are available in the Supplementary Materials (Supplementary Materials S1–S3).

### 2.3 Preparation of CEC-l-OSA hydrogel

To prepare the dynamic hydrogel swelling solution in the same environment as the culture medium of stem cells, CEC (2 wt%, 4.5 ml) and OSA (10 wt%, 0.5 ml) solution were prepared using Dulbecco’s modified Eagles’ medium (DMEM, Gibco) culture medium (10% fetal bovine serum (FBS, Invitrogen), penicillin/streptomycin (Cellgro), respectively. The uniformly dynamic hydrogel could be obtained by homogeneously mixing the above solution at 37°C or under ambient conditions (25°C, 1013.25 kPa) while fixing the equimolar ratio of the reactive groups (M_-NH2_: M_-CHO_ = 1).

### 2.4 Characterization of dynamic behaviors

Macroscopic dynamic behaviors like injection, self-healing, and remodeling of the CEC-l-OSA hydrogel were tested by gel extrusion and incision healing experiments. The microscopic dynamic behavior of the hydrogel and CDHC was monitored by rheological measurements at 37°C (S4).

### 2.5 Storage and transportation of 3D cultured mESCs in hydrogel

The routine culture of mESCs is described in the Supplementary Materials. To fabricate CDHC in which mESCs were 3D encapsulated into the matrix of dynamic hydrogel, mESCs were dissociated to single cells and suspended in DMEM medium containing CEC and OSA under sterile conditions, keeping cell density in the hydrogel was 3 × 10^6^ cell/ml. The CDHC was stored in a sterile and hermetic environment for 3 days and then transferred into a sealed vessel with fresh medium for another 72 h to simulate transportation under ambient conditions. The encapsulated mESCs automatically released from the self-biodegradable hydrogel after the processes of storage and transportation. Collecting the released cells, the cells were subcultured according to the method of routine culture. The first generation of retrieved cells were denoted as S+T+P0, and the cells that continued to culture for 7 and 15 generations were denoted as S+T+P7 and S+T+P15, respectively. Calculation of the cell doubling time of different generations through software (www.doubling-time.com).

### 2.6 Cell viability and immunocytochemistry

Live/dead staining was used to assess cell viability. For immunocytochemistry, the 2D and 3D cultured mESCs in dynamic hydrogel were incubated with primary antibodies including OCT3/4 (1:200, Santa Cruz), NANOG (1:400, Abcam), SSEA-1 (1:200, Santa Cruz) followed by fluorescence secondary antibodies (Supplementary Table S1). Cell nuclei were stained with 4′,6-diamidino-2-phenylindole (DAPI, 1:1000, Sigma). Staining results were observed by laser confocal microscope (S5).

### 2.7 ALP detection and quantitative RT-PCR

Undifferentiated mESCs were identified by both qualitative and quantitative alkaline phosphatase (ALP) analyses. Quantitative RT-PCR reactions for NANOG, Oct3/4, DPPA5α, and Sox2 were performed using the Thermal Cycler Dice Real-Time PCR System (Takara) (S6, S8, Supplementary Table S2).

### 2.8 Statistical analysis

Each experiment was repeated three times. Data were analyzed by Excel (Microsoft) through conducting unpaired and independent Student’s *t*-test for comparison between independent two groups. *p < 0.05* was considered statistically significant.

## 3 Results and discussion

### 3.1 Experiment strategy

**FIGURE 1:**
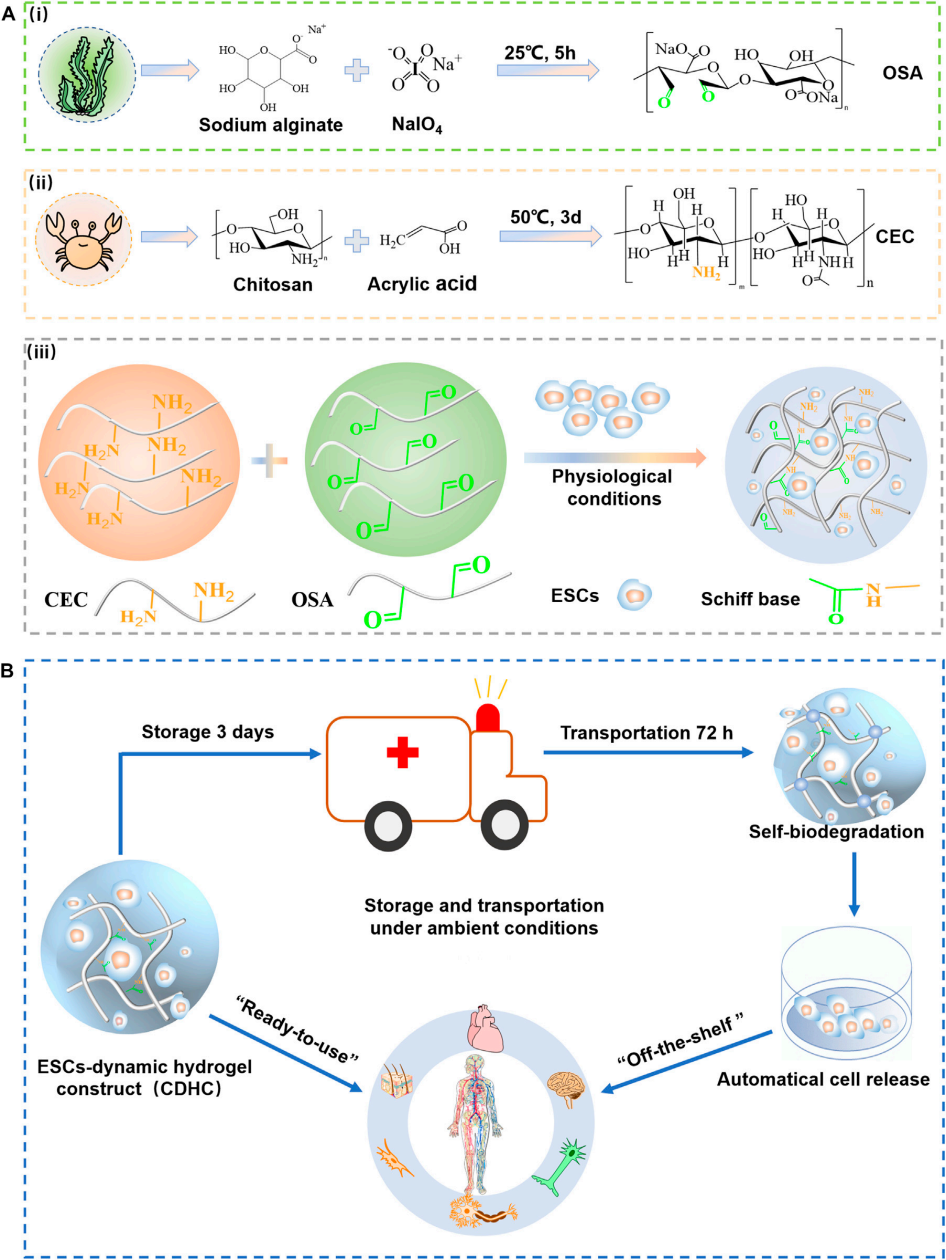
The strategy of storage and transportation of mESCs-dynamic hydrogel construct (CDHC) under ambient conditions. **(A)** Synthesis of **(Ai)** CEC and **(Aii)** OSA, **(Aiii)** fabrication of ESCs-hydrogel construct via encapsulating the cells into CEC-l-OSA hydrogel under physiological conditions. **(B)** The ambient storage and transportation of ESCs-hydrogel construct based on dynamic polysaccharide hydrogel for “off-the-shelf” applications in the biomedical field and research.

The strategy of fabricating “ready-to-use” CDHC in advance, using self-healing hydrogel crosslinked by reversible bonds as mimetics of structurally dynamic ECM microenvironment and soft tissues, and then storage under physiological conditions as an “off-the-shelf” availability for biomedical applications is shown in Figure 1. To *in-situ* build CDHC meeting the actual demand of cell storage and subsequent “ready-to-use” applications, ESCs laden CEC-l-OSA hydrogels were facilely fabricated by mixing a cell suspension, OSA (Figures 1Ai) and CEC (Figures 1Aii) dissolved in the culture medium of stem cells under physiological conditions (pH = 7.4, 37°C). The *in-situ* gelation could be realized by crosslinking polysaccharide backbones via Schiff base reaction between the aldehyde groups from OSA and the amino groups from CEC (Figures 1Aiii). Imide bond is a preferable option to fabricate dynamic hydrogel for assembling ambient storage “ready-to-use” CDHC, because the Schiff base reaction is known as the reversible and biocompatible crosslinking between the polysaccharides, conforming to biomedical applications (Wei et al., 2015; Wei et al., 2016). The ESCs-hydrogel construct could be quickly formed by homogeneous mixing cell suspension and polysaccharide solution for 120 s, which is suitable for homogeneous 3D cell encapsulation. It has been reported that stiff hydrogel hinders the colony growth of encapsulated mESCs, while comparatively, soft hydrogel facilitates the growth of mESC colonies (Ranga et al., 2014; Choi et al., 2016; Marco-Dufort and Tibbitt, 2019), thus, we purposed to fabricate a dynamic hydrogel with low stiffness providing a suitable living microenvironment for ESCs.

Self-biodegradation is important to release mESCs from CDHC. CEC-l-OSA hydrogel crosslinked by imine bonds can self-biodegrade in cell culture medium at 7 days due to a fast hydrolysis rate (Rizwan et al., 2021). In general, pH 4.5 is ideal for the formation of a stable Schiff base. However, the dissociation of the imine bond is stimulated when pH is greater than or less than 4.5. A 3D cell culture was performed under physiological conditions (pH = 7.4). In this case, the hydrogel crosslinked by dynamic imine bond is in a state of slow hydrolysis. Thus, after 6 days of incubation, most cells could be released from the hydrogel. The CDHC was stored in a sealed vessel under an ambient environment for 3 days and then transferred to a fresh medium for another 72 h. The colonies could be harvested after undergoing these processes, owing to partial self-biodegradation of the polysaccharide hydrogel. It is considered that hydrolytic degradation and enzymes produced by the cells may contribute to the degradation of polymer networks.

The synergistic effect of the dynamic hydrogel, including reversibly crosslinked polymer networks and self-biodegradation, provides a dynamic microenvironment and growing space for accommodating mESCs clonal expansion and maintaining pluripotency (Hozumi et al., 2018). After continuous cultivation of 15 generations with passaging released or retrieved cells every 5 days, the mESCs could maintain pluripotency over a long term (25 days), confirmed by ALP staining, immunofluorescence assay of pluripotency (OCT3/4, NANOG, SSEA-1), and RT-PCR analysis of expression marker (NANOG, Dppa5 α, Sox2). The dynamic and self-biodegradable hydrogel provides a tool of facile, cost-effective, and reliable “ready-to-use” CDHC to support short-term ESCs storage and “off-the-shelf” availability, facilitating widespread applications in biomedical applications and research (Figure 1B).

### 3.2 Dynamic behaviors of hydrogel matrix and CDHC

**FIGURE 2:**
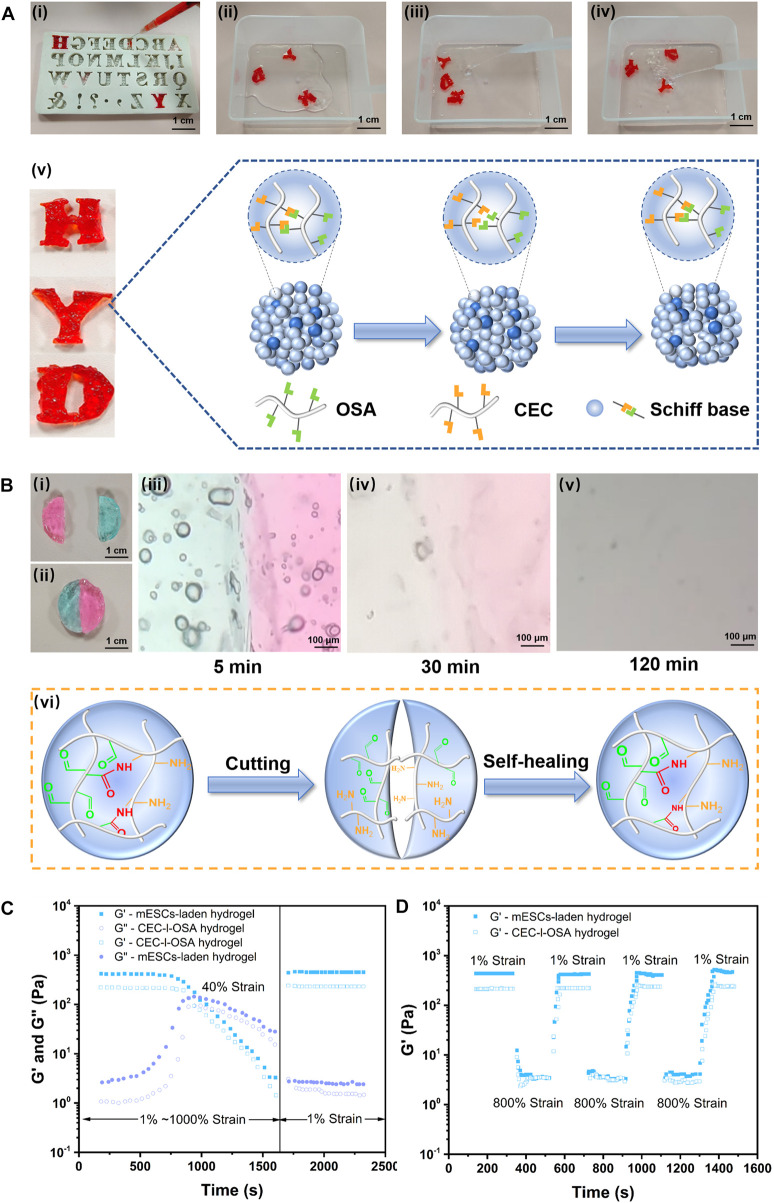
Versatile injectability, remodeling, and self-healing of the dynamic hydrogel and mESCs-hydrogel construct. **(A)** The hydrogel stained with food color red *in-situ* gelation into a syringe and squeezed out into the “H”, “Y”, and “D” templates (Scale bar: 1 cm) **(Ai)**, then the extruded gel particles were self-healed and remodeled to form integrated capitals after 5 min at 37°C without any external intervention (Scale bar: 100 μm) **(Aii)**. The self-healed gel capitals could withstand the impact force generated by water flow without splitting **(Aiii, Aiv)**. **(Av)** Schematic illustration of self-healing and integrity of the remodeled gel capitals constituted by extruded gel particles via dynamic imide bonds. **(B)** Photographs **(Bi, Bii)** (Scale bar: 1 cm), optical microscope images **(Aiii, Aiv, Av)** (Scale bar: 1 cm), and schematic illustrations highlighted dynamic imide bonds **(Avi)** of self-healing of two half disc hydrogels died by methylene blue and rhodamine, respectively. The rheological analysis of the dynamic hydrogel loading or without cells when stain change from 1% to 1000% at 10 rad/s **(C)** and alternating strains (1% and 800%) for three cycles with 200s intervals **(D)**.

The reversible crosslinking mechanism of imide bonds between the aldehyde and amino groups endows the hydrogel with excellent capabilities including injectability, self-healing, and remodeling (Wei et al., 2016). The macroscopic tests were performed to simultaneously evaluate these capabilities. As shown in Figures 2Ai (Supplementary Movie S1), the hydrogel stained with red food coloring was *in-situ* formed into a 26-gauge syringe and then extruded through a needle into a capital template with the shapes of “H”, “Y”, and “D”. The dynamic hydrogel could be facilely squeezed through a needle, owing to easy breakage of dynamic bonds under shear stress during injection. The extruded hydrogel particles automatically self-healed to form new integrities with the same shapes as the capitals, via dynamic imine bonds for 5 min at 37°C without any external interventions (Figures 2Aii). Noteworthy, the self-healed and remodeling hydrogels with capital shapes constituted by gel particles were stable enough to endure PBS flush and maintain their shapes without splitting, demonstrating the self-healed and remodeled hydrogel could withstand the impact force (Figures 2Aiii,iv). A schematic illustration of self-healing and integrity of the remodeled gel capitals constituted by extruded gel particles via dynamic imide bonds is shown in Figures 2Av.

To further macroscopically observe the details of the healing interface, a disc-like piece of the hydrogel was cut into half from the middle (Figures 2Bi), and half dyed methylene blue, half stained with rhodamine, then they were autonomously healed for 5 min under 37°C when combined the two parts with different color (Figures 2Bii). Moreover, the boundary of contacting interface during the healing processes was observed under a microscope at ambient temperature. Initially, the boundary between the different colored pieces was very clear (Figures 2Biii), while the boundary became blurred over time, the interface became a blur and the colors diffused into each other after 30 min (Figures 2Biv). At last, the contacting interface almost disappeared after 120 min. The colors gradually disappeared due to washing out water soluble dyes in PBS solution (Figures 2Bv). These tests demonstrated good injectability and excellent self-healing capability of the CEC-l-OSA hydrogel when exposed to physiological saline (Wang Y. et al., 2022). Figures 2Bvi schematically illustrates the corresponding mechanism of self-healing depending on dynamic imide bonds.

To investigate the effect of 3D loading mESCs on the dynamic and self-healing performances of the hydrogel, we analyzed the rheological behaviors of CDHC, and compared it to a pristine CEC-l-OSA hydrogel (Wei et al., 2015). The rheological analysis in strain amplitude sweep tests at 10 rad/s revealed that, when increasing strain amplitude from 1% to 1000%, the storage modulus (G′) of the as-prepared CDHC significantly descended from 430.83 Pa to 3.28 Pa, whereas the corresponding loss modulus (G″) ascended from 2.64 Pa to 27.98 Pa. Similar, the G′ of the pristine dynamic hydrogel descended from 226.73 Pa to 1.46 Pa, while the corresponding G″ ascended from 1.07 Pa to 15.47 Pa. The results made clear that the mechanical properties of CDHC were higher than pristine hydrogel, meanwhile, the change tendency of G′ and G″ of the CEC-l-OSA hydrogel loading or without cells were similar within the increase of strain amplitude (1%–1000%) (Figure 2C **)**. In addition, when the strain rose from 1% to 40%, and G′ kept at high values, which was significantly higher than the corresponding G″ maintaining at the low lever for both dynamic hydrogel and mESCs-hydrogel construct, indicating elastic-like gel behavior of the associated or crosslinked polysaccharides. Further rising strain to 40%, G′ value began to decrease, meanwhile, G″ value increased with strain growth, and the intersection points of G′ and G″ values of the two hydrogel samples appeared. At this point, the mechanical property of the samples was in the state of gel-to-sol transition, owing to the damage of some reversible imine bonds of polymer networks under relatively high strain. Moreover, when strain continued to rise from 40% to 1000%, most dynamic imide bonds were broken under high strain, leading to translation from the state of gel to sol with the drop of both G′ and G″ values. This liquid-like behavior is derived from the disassociation or uncrosslinking of polysaccharide chains under high stains (Li R. et al., 2022; Liang et al., 2022).

To further confirm the self-healing performance of CDHC, the samples were tested by alternating low (1%) and high (800%) shear strains with 200s intervals and compared with the pristine CEC-l-OSA hydrogel (Figure 2D). In these processes, the hydrogels underwent a transition between the state of gel and sol under alternating shear strains, respectively. When the shear strain rose from 1% to 800%, the CEC-l-OSA hydrogel loading or without cells exhibited the same trend, that is, the G′ values of both samples rapidly decreased dramatically about two orders of magnitude due to shear-thinning. This phenomenon demonstrates that the network structure of the dynamic hydrogels was destroyed under large strain, and the samples underwent a gel-sol transition. The dissociation of imide bonds among polysaccharide chains is believed to contribute to fast stress relaxation of the dynamic covalently crosslinked hydrogel. On the other hand, when the shear strain declined from 800% to 1%, the G′ value almost fully restored to the initial level of gel state instantaneously, as associations or recovery of dissociated dynamic polymer networks.

The above consequences confirm that the excellent self-healing performance of the CEC-l-OSA hydrogel could be maintained when 3D encapsulation of mESCs. Moreover, the G′ value (430.83 Pa) of CDHC was higher than that of the dynamic hydrogel (G′ = 226.73 Pa), which is attributed to the fact that the dynamic hydrogel can wonderfully accommodate the stem cells into dynamic polymer networks after 3D cell encapsulation. The cells take up a certain space in the 3D polymer networks, improving the mechanical strength of the cell-biomaterial construct to a certain extent. The aforementioned fast relaxation, shear-thinning, and self-healing behaviors of CDHC, which is similar to CEC-l-OSA hydrogel, confirmed that the hydrogel encapsulated mESCs inherited self-healing property of the dynamic hydrogel, and the dynamic polysaccharide networks enabled rearrangement to accommodate loading cells.

### 3.3 Storage and transportation of CDHC under ambient condition

**FIGURE 3:**
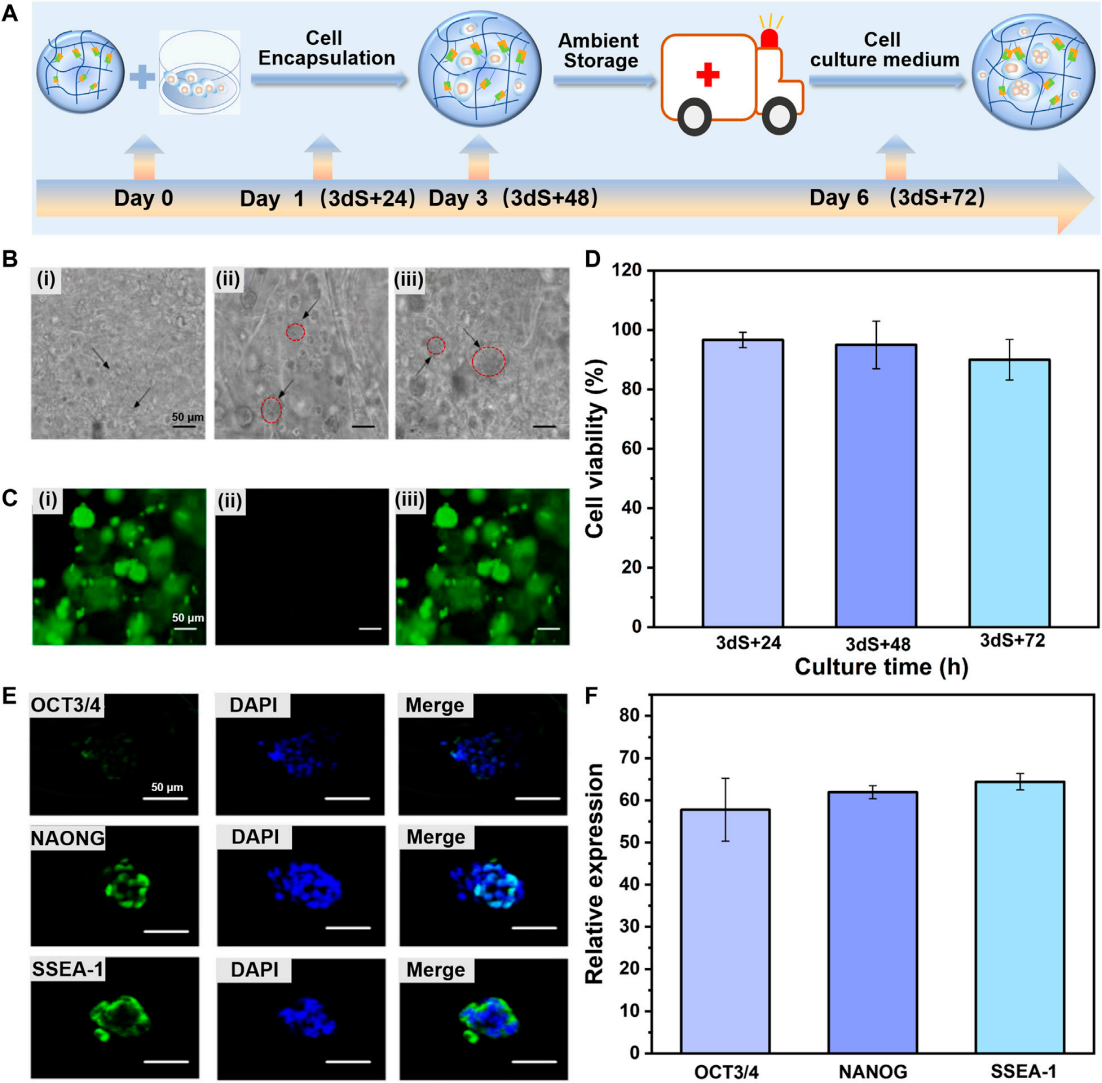
Dynamic hydrogel supports colony expansion and pluripotency of encapsulated mESCs during storage and transportation of mESCs-hydrogel construct at ambient conditions. **(A)** Schematic illustration of the time scale of storage and transportation of mESCs-hydrogel construct. **(B)** Optical microscope images of ESCs encapsulated in hydrogel after storage and then continuous incubation in fresh cell culture medium to transportation for 3 dS+24 h **(Bi)**, 3 dS+48 h **(Bii)**, and 3 dS+72 h **(Biii)** (Scale bar: 50 μm). **(C)** The live/dead staining images of encapsulated mESCs continuously cultured 3 dS+72 h, live cells **(Ci)**, dead cells **(Cii)**, and merge image **(Ciii)** (Scale bar: 50 μm). **(D)** The viability of encapsulated ESCs continuously cultured for 3 dS+24 h, 3 dS+48 h, and 3 dS+72 h. The immunofluorescence staining **(E)** (Scale bar: 50 μm) and relative expression **(F)** for OCT3/4, NAONG, SSEA-1 of encapsulated mESCs continuously cultured for 3 dS+72 h. The data is presented as the means ± SD, n = 12.

To simulate the processes of cell storage, the as-prepared CDHC in which mESCs encapsulated in the CEC-l-OSA hydrogel (3 × 10^6^ cell/ml cells, MEF-conditioned medium containing LIF and mESC specific serum) was stored in a sterile and hermetic environment under ambient temperature for 3 days without adding more culture medium, to reduce the activity of the stem cells and temporarily make cells become quiescent. Subsequently, to simulate cell transportation process, the CDHC was transferred into a sealed vessel filled with fresh cell culture medium and continuously incubated for another 72 h (typically 3 days for domestic delivery) without refreshments under ambient temperature. The corresponding samples during transportation time points of 24, 48, and 72 h was denoted as 3 dS + 24 h, 3 dS + 48 h, and 3 dS + 72 h, respectively. Figure 3A schematic illustration of the time scale of storage and transportation of CDHC. Meanwhile, the colony growth and immunofluorescence staining of pluripotency markers (OCT3/4, NANOG, and SSEA-1) were recorded. The bright-field images showed that mESCs were uniformly distributed within the hydrogel matrix, and colony size increased during the transportation process of continuous incubation after storage. The colonies gradually increased from *c.a.* 5 μm in diameter for 3 dS + 24 h to *c.a.* 60 μm after 3 dS + 72 h transportation, meanwhile, the formation of tight spherical colonies was witnessed, confirming that the dynamic hydrogel promotes robust colony growth during the processes of storage and transportation (Figure 3B). The growth of large and compact colonies was presumably attributed to microenvironment synergistic effects of viscoelastic CEC-l-OSA hydrogel, including dynamic polymer networks, softness, and self-biodegradation, which provide a remodeling surrounding, minimum mechanical interference and stress relaxation.

Furthermore, live/dead staining assay at the transportation time point of 3 dS + 72 h revealed that the majority of mESCs 3D cultured within the dynamic hydrogel adapted and facilitated colony growth, and remained at more than 90% cell viability (acridine orange labels live cells green), whereas very few apoptotic cells (propidium iodide labels dead cells red) (Figure 3C). As shown in Figure 3D, the cell viability was 96.92%, 95.02%, and 90.67% for 3 dS + 24 h, 3 dS + 48 h, and 3 dS + 72 h transportation time points, respectively. The transported stem cells preserved a high survival rate, confirming the cytocompatibility of CEC-l-OSA hydrogel. Pluripotency is the most valuable property to support extensive applications of CDHC. The results of immunofluorescence staining of several representative pluripotency markers demonstrated that substantial expression of OCT3/4, NANOG, and SSEA-1 of the mESCs colony grown within the dynamic hydrogel in 3D cultured for 3 dS + 72 h transportation time point (Figures 3E, F), indicating that the transported stem cells retained the expression of ESCs markers at the protein level, The results showed that the mESCs could express pluripotency marker normally after the processes of encapsulation, storage, and transportation, which proved that 3D culture of the encapsulated mESCs in the dynamic hydrogel could maintain the pluripotency of mESCs well. The above-mentioned results indicate that the dynamic property of polymer networks and transport of sufficient nutrients and oxygen within the self-biodegradable hydrogels could robustly support colony expansion and pluripotency of the 3D encapsulated mESCs during storage and transportation.

### 3.4 Pluripotency of released mESCs from dynamic hydrogel

The ultimate goal of this study is to fabricate CDHC for applications in biomedical fields and research, thus, the features of stem cells like normal proliferation and pluripotency should not be compromised during the period of storage and transportation under ambient conditions (Mei et al., 2010; Celiz et al., 2014). To this end, the properties of mESCs released from dynamic hydrogel were analyzed. Indeed, in our system, the encapsulated stem cells could be automatically released from the CEC-l-OSA hydrogel after the processes of 3 days of storage and further 72 h transportation, owing to the self-biodegradation of the dynamic hydrogel.

**FIGURE 4:**
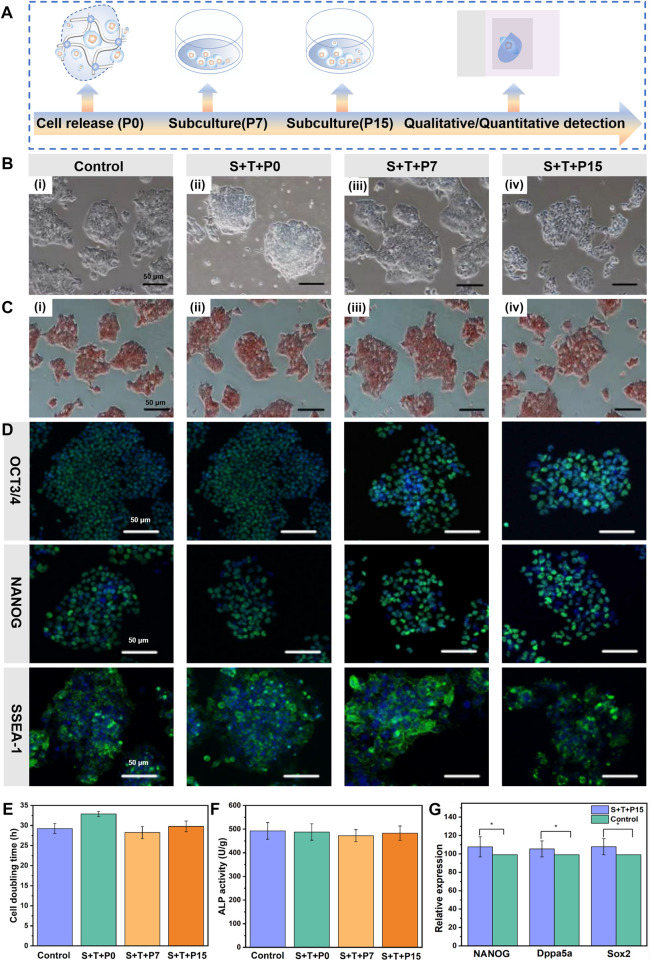
Pluripotency of automatically released mESCs from the dynamic hydrogel. **(A)** Schematic illustration of the time scale of continuous subculture and quantitative detection of released mESCs. **(B)** Optical microscope images, **(C)** ALP staining images, **(D)** Immunofluorescence staining of routinely cultured ESCs without hydrogel encapsulation and before storage/transportation **(Bi)**, S+T+P0 **(Bii)**, S+T+P7 **(Biii)**, and S+T+P15 **(Biv)** (Scale bar: 50 μm). **(E)** Cell doubling time, **(F)** ALP activity, and **(G)** Relative expression for NANOG, Dppa5a, Sox2 of routinely cultured ESCs without hydrogel encapsulation and before storage/transportation (control) and S+T+P15. The data are presented as the means ± SD, n = 12, *p < 0.05.

Based on the capabilities of automatical cell release and self-biodegradation, mESCs could be facilely harvested or retrieved from hydrogel after transportation within a certain period, without using any enzymes or external stimuli. These merits facilitate to obtain of a biocompatible “off-the-shelf” construct of ESCs-dynamic hydrogel. To further evaluate the proliferation and stemness of released mESCs during long-term culture, the behaviors of doubling time (the time of doubling cell number of its initial value) and pluripotency of the mESCs automatically released from the hydrogel after 3 days storage and 120 h transportation, was evaluated after further incubation. The morphology and doubling time of the mESCs just released from hydrogel (denoted as S+T+P0), and the cells after continued culture for 7 and 15 generations (denoted as S+T+P7, S+T+P15) with cell passaging every 5 days were characterized. The results showed that the stem cells released from hydrogel, both freshly released S+T+P0 cells (Figures 4Aii) and continuously cultured for S+T+P7 (Figures 4Aiii) and S+T+P15 (Figures 4Aiv) generations, could form spherical and compact colonies and had no obvious morphological difference comparing with the stem cells cultured by routine culture (control group) (Figures 4Ai). At the same time, the released stem cells were still enabled to proliferate with culture time, the doubling time was 32.87%, 28.24%, and 29.77%, respectively, for S+T+P0, S+T+P7, S+T+P15 generations which were not significantly different from that of routinely cultured mESCs (29.25%) under the same cell seeding density (Figure 4E). The above results proved that the retrieved mESCs undergone storage and transportation can not only sustain proliferation but also ensure a high biological activity to form colonies, that is, at the same level as that of routinely cultured mESCs.

To judge whether the mESCs automatically released from the hydrogel still retained pluripotency, ALP staining, a specific and sensitive method for phenotypic assessment of ESCs by determination of the activity of alkaline phosphatase, was used to preliminarily detect the pluripotency of released stem cells. As shown in Figure 4C, the retrieved mESCs (S+T+P0) and the stem cells after continuing to culture as S+T+P7 and S+T+P15 generations were stained brown-red under the action of ALP reagent, like routinely cultured stem cells without hydrogel encapsulation and before storage/transportation. The quantitative test of ALP staining results further showed that there was no significant difference in the activity of alkaline phosphatase between the retrieved mESCs and routinely cultured stem cells without hydrogel encapsulation and before storage/transportation (Figure 4F). The above results preliminarily proved that the released stem cells could resume normal capabilities of colony formation and pluripotency.

To further demonstrate the retrieved mESCs could maintain pluripotency after long-term subculture, the stem cell markers both in protein and mRNA levels were analyzed. The compact and well-defined colonies formed by the stem cells with S+T+P0, S+T+P7, and S+T+P15 generations when cultured for 48 h, and the colonies exhibited robust immunofluorescence staining for Oct3/4, NANOG, and SSEA-1 (Figure 4D). At the same time, there was no significant difference in their expression levels compared with the routine passaging of mESCs. To quantitatively verify the pluripotency of the released mESCs with long-term passaging, we next analyzed the expression of pluripotency of related genes S+T+P15 generation using RT-PCR detection. Consisting with the results of immunofluorescence staining, the RT-PCR analysis also showed significantly higher expression levels of NANOG, Dppa5a, and Sox2, and there was no significant difference in the expression compared with routinely passaged stem cells, indicating released mESCs maintained functional pluripotency after 25 days continuous culture (Figure 4F). In Figure 4G, the S+T+P15 group was the mESCs automatically released from the dynamic hydrogel after 3 days of storage and 120 h transportation, and continued culture for 15 generations (denoted as S+T+P7, S+T+P15). Due to the dynamic hydrogel that could support the self-renewal and differentiation of ESCs in biomimetic 3D and dynamic environments, the S+T+P15 group has higher expression of NANOG, Dppa5a, Sox2 than the routinely cultured ESCs without hydrogel encapsulation and before storage/transportation (control group). Due to the dynamic hydrogel that could support the self-renewal and differentiation of ESCs in biomimetic 3D and dynamic environments (Xu et al., 2022), these findings together indicate that the CEC-l-OSA hydrogel could be an effective dynamic culture platform for the ESCs-hydrogel construct that can keep the pluripotency of stem cells at both protein and gene levels.

## 4 Conclusion

The approaches of safe storage and transportation of ESCs for reliable preservation of stem cell survival and pluripotency is a bottleneck to the distribution of stem cells for biomedical applications and research. However, the current cryopreservation involves ice crystal formation, toxic cryoprotective agents, and cold chain management, which are technically challenging and financially expensive. The goal of this study is to design a facile and efficient approach for the storage and transportation of ESCs under ambient conditions. To this end, we proposed an alternative approach taking into consideration of ambient storage, transportation, and “off-the-shelf” availability simultaneously. That is, storage and transportation of stem cells facilely in the form of CDHC, through encapsulating ESCs within dynamic and self-biodegradable polysaccharide hydrogel. Conclusions could be drawn from the results that our facile and economic approach only involves *in-situ* encapsulation of stem cells inside dynamic polysaccharide hydrogel, storage, and transportation for a few days under ambient conditions. The large and tight spherical colonies with an expression of pluripotency markers (OCT3/4, NANOG, and SSEA-1) formed in dynamic hydrogel after storage and subsequently transformation, and the viability of mESCs remained >90%. Moreover, the automatically released stem cells resumed typical capabilities of colony formation and pluripotency after long-term continuous culture (25 days) confirmed by stem cell markers both in protein and mRNA (NANOG, Dppa5a, and Sox2) levels. This preservation approach of ESCs-hydrogel construct is easy to scale up for generation, storage, and transportation of a large number of biocompatible and self-biodegradable ESCs-hydrogel construct under ambient conditions, having wide potentials in biomedical applications and research as “off-the-shelf” availability.

## Data Availability

The datasets presented in this study can be found in online repositories. The names of the repository/repositories and accession number(s) can be found in the article/Supplementary Material.
