# Supplementary material for: Dynamic and self-biodegradable polysaccharide hydrogel stores embryonic stem cell construct under ambient condition
Source: Front Bioeng Biotechnol. 2023 May 11;11:1169124. doi: 10.3389/fbioe.2023.1169124 (PMC10219609; doi:10.3389/fbioe.2023.1169124)
Supplement: Supplementary file 1 [file DataSheet1.PDF]

Supplementary Material

Dynamic and self-biodegradable polysaccharide hydrogel stores embryonic stem cell construct under ambient condition

Kuan Yang, Wei Wei, Li Ting Gao, Xin Yi Zhao, Zhenqi Liu, Jianhui Li, Haopeng Li, Hideyuki Miyatake, Yoshihiro Ito, Yong Mei Chen*

***Correspondence:** Yong Mei Chen: chenyongmei@sust.edu.cn

S 1 Synthesis and characterization of CEC

CEC was synthesized through Michaels addition reaction according to our previously reported method (Wei et al., 2015). Briefly, 6.2 mmol chitosan was dissolved in an aqueous solution containing 21.3 mmol acrylic acid under stirring for 3 days at 50 °C. Subsequently, adjust the pH of above solution to 10-12 by dropping 1 M NaOH. Then, the solution was dialyzed in deionized water which was changed twice 3 days, using the dialysis bag with 8000Da interception molecular weight. Last, CEC powder could be obtained by freeze-drying. The ^{1}H-NMR was performed to confirm the successful preparation of CEC. The 10 mg dry products dissolved in 0.5 ml D_2O were tested by 400 MHz nuclear magnetic resonance instrument. The degree of substitution of amino group in the product is 48% by comparing the peak areas of the acetamide methyl protons (δ=1.94) of chitosan and methylene protons (δ=2.83) of acrylic acid in CEC (1.94 (s, 3 H, COCH3), 2.83 (s, 2 H, CH2CO2Na), 3.30~4.87 (m, glucosamine).

S 2 Synthesis and characterization of OSA

The OSA was synthesized by oxidation of sodium periodate reported in our previous work (Zheng et al., 2021). Firstly, magnetically stirring 100 ml aqueous

solution dissolved 5 mmol sodium alginate and 5 mmol sodium periodate ($NaIO_4$) in the dark at 25 °C for 5 h. Then, adding 1.5 mL ethylene glycol and stirred for 1.0 h to terminate the reaction. OSA powder could be obtained after placing the product in dialysis bags (MWCO 3000) and soaking it in distilled water for 3 days with changed water every day, and the final lyophilization of dialysate overnight. The oxidation degree of OSA is 50% determined by hydroxylamine hydrochloride titration. The detailed information was shown in supporting information.

S 3 Detection of oxidation degree of oxidized sodium alginate

The oxidation degree of oxidized sodium alginate (OSA) was determined by iodometry, by determining the content of unconsumed periodate after the oxidized reaction. Neutralization of reaction mixture (5 mL) by adding 10 mL of 10 wt% sodium bicarbonate solution. Then, 20 mL of 20% potassium iodide solution was added. After reacting the above solution in the dark at 25 °C for 30 min, the liberated iodine was titrated with standardized sodium thiosulphate solution (0.01 mol/L) using starch (1 wt%) as the indicator. According to three parallel tests, the oxidation degree of OSA in this experiment was calculated as 50%.

S 4 Characterization of dynamic behaviors

Macroscopic dynamic behaviors like injection, self-healing and remodeling of the CEC-l-OSA hydrogel was tested by extruding disc-shaped hydrogels into a capital template with the shape of “H”, “Y”, and “D”, and then cultured for 5 min at 37 °C without any external intervention. The hydrogel capitals constituted by extruded gel particles were taken out from the template and immersed in PBS (pH=7.4). Their

stability was observed by flushing using PBS solution. In addition, self-healing phenomenon was also tested by an incision healing experiment.

Microscopic dynamic behavior of the hydrogel and CDHC (diameter: 15 mm, thickness: 20mm) was monitored by rheological measurements at 37 °C. In strain amplitude sweep test, the storage modulus (G′) and loss modulus (G′′) of samples were recorded when angular velocity was fixed at 10 rad/s, while changing strain (γ) in the range of 10%-1000%. The alternate step strain sweep was tested at a fixed angular frequency (10 rad/s) while switching amplitude oscillatory strains from small strain (γ = 1.0%) to subsequent large strain (γ = 800%) with 200 s for every strain interval.

S 5 Routine culture of mESCs

Gelatin-coated culture dishes were prepared by incubation of gelatin solution (0.1%, Millipore) for 2 h. The mESCs, kindly presented by prof. Jian Kang Liu from School of Life Science and Technology and Frontier Institute of Life Science, Xi'an Jiaotong University, were cultured on the gelatin-coated culture dishes using cell culture medium containing 15% KnockOut^{TM}-Serum Replacement (Gibco), 1×GlutaMAX^{TM}-I supplement (Gibco), 1×MEM non-essential amino acid solution (Gibco), 1×penicillin/streptomycin, 0.1 mM β-mercaptoethanol (MP biomedical) and 1000 U ml^{-1} ESGRO® mLIF (Millipore) in DMEM. The cells were trypsinized and routinely passaged every 2-3 days. All cultures were performed at 37 °C in a humidified atmosphere of 5% CO_2. The culture medium was changed every day.

S 6 Cell viability test and immunocytochemistry

Live/dead staining was used to assess cell viability, living cells were labeled with green color Calcein AM (Gibco), and apoptotic cells were labeled with red color EthD-1 (Gibco). After rinsing with PBS, the 2D and 3D mESCs cultured in dynamic hydrogel were incubated with 2 μM Calcein AM and 4 μM EthD-1 in PBS for 25 min.

Subsequently, the samples were washed with PBS 3 times and observed under fluorescence microscope. All assays were done with three parallel samples. The samples of immunocytochemistry were washed with PBS and fixed with 4% paraformaldehyde for 20 min. The PBS rinsed samples were permeabilized with 0.20% Triton-X 100 (Amresco) and incubated with 5% bovine serum albumin (BSA, Sigma) for 6 h before tests.

S 7 The information on fluorescence secondary antibodies

Table S1 Fluorescence secondary antibodies and manufacturers

Antibody name	Manufacturer
Oct-4A (C30A3) Rabbit mAb Antibody	Cell Signaling Technology
NANOG(D2A3) XP® Rabbit mAb (Mouse Specific) Antibody	Cell Signaling Technology
SSEA-1 (MC480) Mouse mAb Antibody	Cell Signaling Technology
Anti-mouse IgG (H+L), F(ab)2 Fragment (Alexa Fluor® 488 Conjugate)	Cell Signaling Technology
Anti-rabbit IgG (H+L), F(ab)2 Fragment (Alexa Fluor® 488 Conjugate)	Cell Signaling Technology

S 8 ALP detection

Both qualitative and quantitative ALP analyses were carried out post cell culture. In the case of qualitative characterization, ALP detection kit (Sigma-Aldrich) was used for ALP staining. Briefly, mESCs were fixed in 4% paraformaldehyde solution for 20 min, then rinsed 3 times by PBS and immersed in staining solution for 30 min in the dark at room temperature. Cells were rinsed and covered by a counterstain solution for 2 min, then rinsed again and stored in PBS in the dark prior to microscopy analysis. The ALP activity was quantitatively analyzed by another ALP assay kit (Nanjing Jiancheng) according to the manufacturer's instructions. After rising by PBS, mESCs

samples were soaked in 250 L RIPA lysate (Five Heart) at 4 ℃ for overnight. The acquired supernatant was assessed by the ALP assay kit and a BCA protein assay kit (Five Heart) to get the ALP activity per unit mass of protein which can represent the ALP activity of a single cell.

Information on primer sequences

Table S2. Primer sequences used in quantitative RT-PCR

Gene name	Primer sequences
NANOG	F: 5′-TTGCTTACAAGGGTCTGCTACT-3′<br>R: 5′-ACTGGTAGAAGAATCAGGGCT-3′
Dppa5α	F: 5′-TCGGGCTAAATGGATGCTTCA-3′<br>R: 5′-ACAAGGACTGGAAACTGGCTTCAC-3′
Sox-2	F: 5′-AACCGATGCACCGCTACGA-3′<br>R: 5′-TGCTGCGAGTAGGACATGCTG-3′

Move S1

The macroscopic tests were performed to simultaneously evaluate the capabilities of injectability, remodeling and self-healing as follows. (i) The CEC-l-OSA hydrogel stained with food coloring red was *in-situ* formed into a 26-gauge syringe and then extruded through a needle into a capital template with the shape of “H”, “Y”, and “D”. (ii) The extruded hydrogel particles automatically self-healed to form new integrities with the same shapes as the capitals, via dynamic imine bonds for 5 min at 37 °C without any external interventions. (iii) The self-healed and remodeling hydrogels with capital shapes constituted by gel particles were stable enough when flushing to PBS solution, and the hydrogel capitals could maintain their shape without splitting, demonstrating the self-healed and remodeled hydrogel could withstand the impact force generated by a flush with PBS solution.
